# Impact of Rheumatoid Arthritis on Postoperative Outcomes Following Lumbar Spine Surgery: A Systematic Review and Meta-Analysis

**DOI:** 10.7759/cureus.98150

**Published:** 2025-11-30

**Authors:** Shahmeen Rasul, Rana Ahmed, William J Austin, Osasenaga Bencharles, Shashwat Shetty, Arnov Mukherjee, Siddhesh V Kulkarni, Danish Allahwala

**Affiliations:** 1 Trauma and Orthopedics, University Hospitals of Derby and Burton (UHDB) NHS Foundation Trust, Burton-on-Trent, GBR; 2 Emergency Medicine, The Hillingdon Hospitals NHS Foundation Trust, Uxbridge, GBR; 3 Orthopedics, University Hospitals of Derby and Burton (UHDB) NHS Foundation Trust, Derby, GBR; 4 Medicine and Surgery, University Hospitals of Derby and Burton (UHDB) NHS Foundation Trust, Derby, GBR; 5 Orthopedics, The Hillingdon Hospitals NHS Foundation Trust, Uxbridge, GBR; 6 Trauma and Orthopedics, Queen's Hospital Burton, Burton-on-Trent, GBR; 7 Trauma and Orthopedics, The Hillingdon Hospitals NHS Foundation Trust, Uxbridge, GBR; 8 Nephrology, Fatima Memorial Hospital, Karachi, PAK

**Keywords:** lumbar spine surgery, postoperative complications, reoperation, rheumatoid arthritis, surgical site infection

## Abstract

This systematic review and meta-analysis critically evaluated the impact of rheumatoid arthritis (RA) on postoperative outcomes following lumbar spine surgery. A comprehensive literature search was performed across multiple electronic databases from January 2010 to October 2025. Studies involving adult patients with confirmed RA undergoing lumbar spine surgery with comparator groups were included. Eight retrospective cohort studies representing diverse populations were analyzed. Pooled analysis revealed that patients with RA had a significantly higher risk of reoperation compared to non-RA patients, with a relative risk (RR) of 1.34 and a 95% confidence interval (CI) of 1.15-1.57, though high heterogeneity was noted. Surgical site infection analysis demonstrated a 45% increased risk in RA patients with an RR of 1.45 and a 95% CI of 1.28-1.65, with no heterogeneity observed. Other clinical outcomes, including mortality, pneumonia, acute kidney injury, and sepsis, showed no statistically significant differences between the groups. Patients with RA face significantly elevated risks of reoperation and surgical site infection following lumbar spine surgery, likely due to compromised bone quality, impaired healing capacity, and immunosuppressive medications. These findings have important implications for preoperative counseling and perioperative risk stratification. Future prospective research with detailed characterization of disease activity and medication use is needed to facilitate individualized risk assessment.

## Introduction and background

Lumbar spinal disorders, including degenerative disc disease, spinal stenosis, and spondylolisthesis, represent a significant burden on healthcare systems worldwide, affecting millions of individuals and often requiring surgical intervention when conservative management fails [1]. These conditions are among the most common reasons for spinal surgery, with increasing incidence rates observed in aging populations [2]. Concurrently, rheumatoid arthritis (RA), a chronic systemic autoimmune disease characterized by inflammatory polyarthritis, affects approximately 0.5%-1% of the global population and frequently coexists with spinal pathology [3]. The prevalence of RA continues to rise, particularly among older adults who are also at higher risk for degenerative spinal conditions [4].

Patients with RA present unique challenges in the perioperative setting due to the systemic nature of their disease. The chronic inflammation associated with RA affects multiple organ systems and may compromise bone quality, immune function, and wound healing capacity [5]. Furthermore, long-term use of disease-modifying antirheumatic drugs (DMARDs), corticosteroids, and biological agents can significantly alter immune responses and tissue repair mechanisms [6]. The cervical spine involvement in RA, particularly atlantoaxial instability, has been extensively studied; however, the impact of RA on lumbar spine surgery outcomes remains inadequately characterized [7]. While cervical manifestations of RA are well-documented, the implications for subaxial and lumbar spine procedures require further investigation.

Existing literature suggests that RA patients undergoing orthopedic procedures may experience higher rates of postoperative complications, including surgical site infections, delayed wound healing, and increased length of hospital stay [8]. Several studies have reported elevated complication rates in RA patients following joint arthroplasty procedures [9]. However, data specific to lumbar spine surgery remain limited and inconsistent. Some studies report comparable outcomes between RA and non-RA patients, while others demonstrate significantly elevated complication rates and poorer functional recovery [10,11]. This heterogeneity in findings necessitates a comprehensive synthesis of available evidence.

Understanding the impact of RA on postoperative outcomes following lumbar spine surgery is crucial for several reasons. First, it enables surgeons to provide accurate preoperative counseling regarding risks and expected outcomes. Second, it facilitates the development of targeted perioperative protocols to optimize outcomes in this high-risk population [12]. Third, it informs decision-making regarding the timing of surgery relative to RA disease activity and medication management [13]. Risk stratification and individualized treatment planning are essential components of modern surgical care.

This systematic review and meta-analysis aim to critically evaluate and synthesize existing literature to determine whether RA independently affects postoperative complications, functional outcomes, reoperation rates, and mortality following surgical intervention for lumbar spinal disorders. By providing robust evidence synthesis, this study will inform clinical practice and identify areas requiring further research.

## Review

Methodology

The present study was conducted according to guidelines of Preferred Reporting Items for Systematic Reviews and Meta-Analyses (PRISMA) [14].

Literature Search and Search Strategy

A comprehensive systematic literature search was conducted across multiple electronic databases, including PubMed/MEDLINE, Embase, Cochrane Central Register of Controlled Trials, Web of Science, and Scopus, from inception to from January 1, 2010, to October 15, 2025. The search strategy employed a combination of Medical Subject Headings terms and free-text keywords related to RA, lumbar spine disorders, and surgical outcomes.

The search strategy included the following key terms and their variations: "rheumatoid arthritis" OR "RA" OR "inflammatory arthritis" AND "lumbar spine" OR "lumbar vertebrae" OR "lumbosacral spine" OR "spinal stenosis" OR "degenerative disc disease" OR "spondylolisthesis" OR "spinal fusion" OR "laminectomy" OR "discectomy" OR "decompression" AND "surgery" OR "surgical" OR "operative" OR "postoperative" AND "outcomes" OR "complications" OR "mortality" OR "morbidity" OR "reoperation" OR "infection" OR "functional outcome". The search strategy was adapted appropriately for each database, considering their specific indexing systems and controlled vocabularies.

No language restrictions were imposed, and articles in languages other than English were translated when necessary. Gray literature was searched through conference proceedings, dissertation databases (ProQuest), and clinical trial registries (ClinicalTrials.gov and WHO International Clinical Trials Registry Platform). Additionally, manual searching of reference lists of included studies and relevant systematic reviews was performed to identify any additional eligible studies.

Study Selection

Two independent reviewers screened all identified titles and abstracts using predefined eligibility criteria. Any discrepancies were resolved through discussion or involvement of the principal investigator. Studies were included if they involved adult patients (≥18 years) with a confirmed diagnosis of RA undergoing any type of lumbar spine surgery, including spinal fusion, decompression, laminectomy, discectomy, or instrumentation, with a comparator group of patients without RA undergoing similar lumbar spine procedures.

Studies were excluded if they were case reports or case series with fewer than 10 patients and did not report comparative data between RA and non-RA patients, focused exclusively on cervical or thoracic spine surgery, were animal studies, in vitro studies, reviews, editorials, or commentaries. Full-text articles of potentially eligible studies were retrieved and independently assessed by two reviewers against the eligibility criteria. A PRISMA flow diagram was constructed to document the study selection process, including the number of records identified, screened, excluded with reasons, and finally included in the qualitative synthesis and quantitative meta-analysis.

Data Extraction

A standardized, piloted data extraction form was developed using Microsoft Excel (Microsoft Corporation, Redmond, WA). Two independent reviewers extracted data from all included studies, with discrepancies resolved through discussion or consultation with a third reviewer. Data extracted from included studies were the first author's name and year of publication, country of origin, study design, sample size, mean age, gender distribution, and outcome measures. Outcomes assessed in this study included reoperation, surgical site infection, and other complications.

Quality Assessment

The methodological quality and risk of bias of included studies were assessed independently by two reviewers using the Newcastle-Ottawa Scale (NOS) for observational studies [15]. The NOS evaluates three domains: selection of study groups (representativeness of exposed cohort, selection of nonexposed cohort, ascertainment of exposure, and demonstration that outcome of interest was not present at start of study), comparability of groups (comparability on the basis of design or analysis), and ascertainment of outcome (assessment of outcome, adequate follow-up length, and adequacy of follow-up). Studies can be awarded a maximum of nine stars, with higher scores indicating better quality.

Data Analysis Plan

All statistical analyses were performed using Review Manager (RevMan) version 5.4 software (The Cochrane Collaboration, London, UK). For dichotomous outcomes, including reoperation, surgical site infection, mortality, pneumonia, acute kidney injury, and sepsis, relative risks (RRs) with 95% CIs were calculated as the primary effect measures. Statistical heterogeneity among studies was assessed using the chi-square test and quantified using the I² statistic. I²​​​​​​​ values of 25%, 50%, and 75% were considered to represent low, moderate, and high heterogeneity, respectively. Forest plots were generated to visually display individual study results and pooled effect estimates. Statistical significance was set at a p value of less than 0.05 for overall effect estimates. Due to the limited number of included studies for each outcome, formal assessment of publication bias could not be conducted.

Results

**Figure 1:**
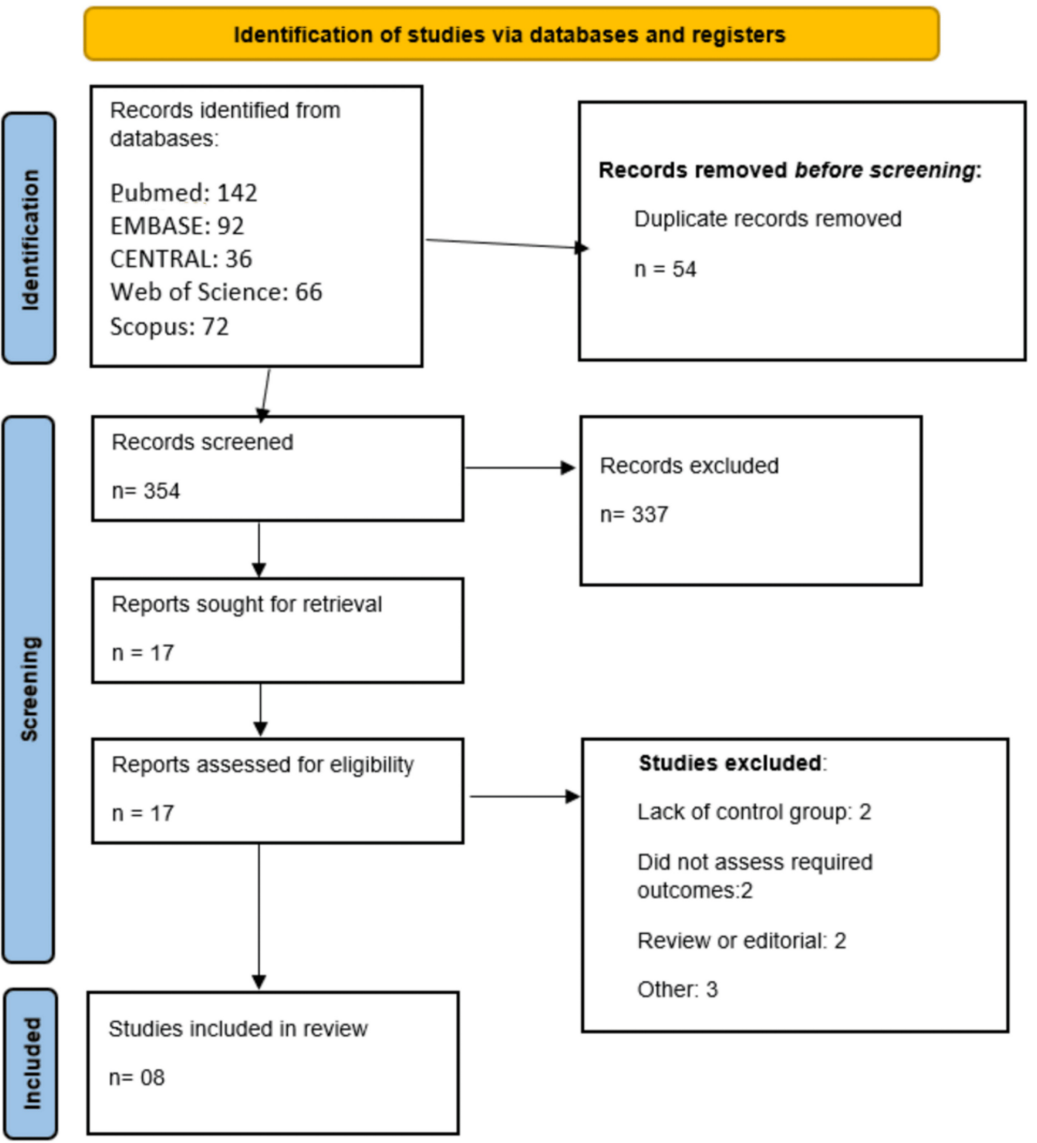
Study selection process

Through initial database screening, 408 studies were found. After removing duplicates, studies were initially screened using their title and abstracts. Full text of 17 studies was retrieved, and detailed screening was done based on predefined inclusion and exclusion criteria. A total of eight retrospective cohort studies were included, representing diverse populations from the United States, China, Korea, France, and Norway (Figure 1). Table 1 presents demographic characteristics of included studies. Sample sizes varied considerably across studies, ranging from small single-center cohorts to large nationwide datasets. The mean age of patients with RA was approximately mid-50s to late-60s across studies, largely comparable to their respective control groups. Table 2 presents the quality assessment of the included studies.

**Table 1:** Characteristics of included studies (n = 8) RA: rheumatoid arthritis

Study	Year	Study design	Region	Groups	Sample size	Age (years)	Males (n)
Bell et al. [16]	2021	Retrospective cohort	United States	RA	3,021	NA	2,227
				Control	44,186	NA	32,714
Gulati et al. [17]	2016	Retrospective cohort	Norway	RA	37	68.2	9
				Control	1,396	67.7	721
Jain et al. [18]	2025	Retrospective cohort	United States	RA	34	68.7	7
				Control	204	62.5	50
Kang et al. [19]	2016	Retrospective cohort	Korea	RA	40	64.3	1
				Control	134	65.3	17
Park et al. [20]	2022	Retrospective cohort	Korea	RA	2,239	64.5	603
				Control	11,195	64.5	3,033
Ratnasamy et al. [21]	2023	Retrospective cohort	United States	RA	2,149	56.4	781
				Control	8,485	56.4	3,087
Thiébaut et al. [22]	2021	Retrospective cohort	France	RA	13	57.4	4
				Control	36	54.2	13
Ye et al. [23]	2025	Retrospective cohort	China	RA	8,992	64	2,297
				Control	17,984	64	4,612

**Table 2:** Quality assessment of included studies

Study	Selection	Comparability	Outcome	Overall
Bell et al. [16]	3	1	3	Good
Gulati et al. [17]	3	2	3	Good
Jain et al. [18]	3	2	3	Good
Kang et al. [19]	4	2	3	Good
Park et al. [20]	4	2	3	Good
Ratnasamy et al. [21]	4	2	3	Good
Thiébaut et al. [22]	3	2	3	Good
Ye et al. [23]	4	2	3	Good

Reoperation

**Figure 2:**
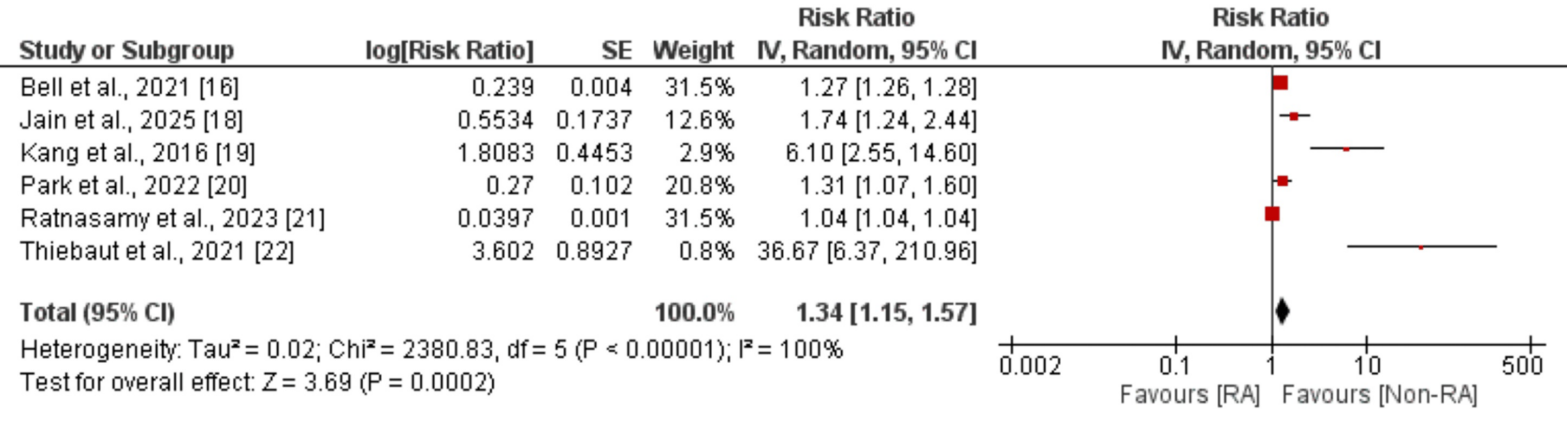
Comparison of risk of reoperation SE: standard error; IV: intravenous; CI: confidence interval; RA: rheumatoid arthritis Source: [16,18-22]

Five studies were used in the pooled analysis comparing the risk of reoperation between patients with and without RA undergoing spine surgery, and the results are shown in Figure 2. Pooled analysis showed that the risk of reoperation was significantly higher in patients with RA compared to non-RA (RR: 1.34, 95% CI: 1.15-1.57). High heterogeneity was reported among the study results.

Surgical Site Infection

**Figure 3:**
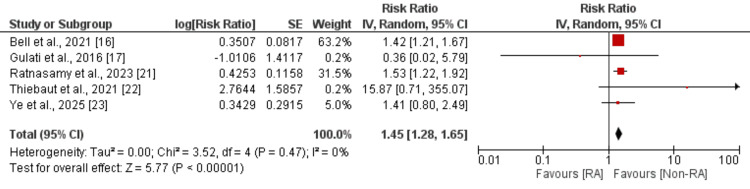
Comparison of surgical site infection SE: standard error; IV: intravenous; CI: confidence interval; RA: rheumatoid arthritis Source: [16,17,21-23]

Five studies compared the risk of surgical site infection between patients with and without RA undergoing spine surgery, and the results are shown in Figure 3. Pooled analysis showed that the risk of surgical site infection was significantly higher in subjects with RA compared to non-RA (RR: 1.45, 95% CI: 1.28-1.65). No heterogeneity was reported among the study results (I²​​​​​ = 0%).

Other Complications

Comparison of clinical outcomes between patients with RA and non-RA controls showed no statistically significant differences across the evaluated endpoints. The risk of death was similar between groups (RR: 0.96; 95% CI: 0.69-1.32; I² = 14%), indicating no clear increase in mortality among RA patients. Higher but imprecise risk estimates were observed for pneumonia (RR: 1.94; 95% CI: 0.70-5.36; I² = 97%), acute kidney injury (RR: 1.52; 95% CI: 0.81-2.85; I² = 96%), and sepsis (RR: 1.97; 95% CI: 0.95-4.12; I² = 94%), though wide CIs and substantial heterogeneity limit certainty in these findings. Overall, the meta-analysis did not demonstrate a significant excess risk of major complications among patients with RA compared with non-RA individuals (Table 3).

**Table 3:** Other complications RR: risk ratio; CI: confidence interval

Outcome	RR (95% CI)	I^2^
Death	0.96 (0.69-1.32)	14%
Pneumonia	1.94 (0.70-5.36)	97%
Acute kidney injury	1.52 (0.81-2.85)	96%
Sepsis	1.97 (0.95-4.12)	94%

Discussion

This systematic review and meta-analysis represents the comprehensive synthesis of evidence examining the impact of RA on postoperative outcomes following lumbar spine surgery. Our analysis of eight retrospective cohort studies involving diverse populations across multiple countries revealed that patients with RA face significantly elevated risks of reoperation and surgical site infection compared to their non-RA counterparts, while mortality and major medical complications showed no statistically significant differences between groups.

The most clinically significant finding of our study was the 34% increased risk of reoperation in RA patients. Meta-analysis conducted by Honda et al. [24] also reported a higher incidence of reoperations in RA patients compared to non-RA subjects. A large registry-based study by Jämsen et al. examining total joint arthroplasty outcomes reported similarly elevated revision rates in RA patients compared to osteoarthritis patients, attributing this to compromised bone quality and ongoing systemic inflammation [9]. The biological mechanisms underlying increased reoperation risk in RA patients likely involve multiple factors, including osteoporosis secondary to chronic inflammation and corticosteroid use, impaired bone healing capacity, and the potential for progressive spinal instability in the setting of systemic disease [25]. Furthermore, the chronic use of DMARDs and biologics may interfere with the bone remodeling processes essential for successful spinal fusion, as demonstrated by Goodman et al. in their investigation of perioperative medication management [12]. The present study reported high heterogeneity in this outcome, possibly because of differences in surgery, study population, duration of follow-up, and characteristics of the sample. However, due to the limited number of studies, we were unable to perform subgroup analysis.

Our finding of a 45% increased risk of surgical site infection (RR: 1.45, 95% CI: 1.28-1.65) in RA patients is particularly noteworthy given the absence of heterogeneity (I² = 0%), suggesting a consistent effect across diverse populations and healthcare settings. This result corroborates earlier work by Bongartz et al., who reported significantly elevated infection rates following total hip and knee arthroplasty in RA patients [8]. A systematic review by Cordtz et al. examining infection risk across various surgical procedures in RA patients found a pooled RR of 1.56 (95% CI: 1.38-1.77) for postoperative infections, remarkably consistent with our findings [26]. The heightened infection susceptibility in RA patients reflects the complex interplay between disease-related immune dysregulation and iatrogenic immunosuppression from therapeutic agents. Tumor necrosis factor inhibitors and other biologic agents, while effective in controlling RA disease activity, substantially increase infection risk by compromising the host immune response [27].

The substantial heterogeneity observed for medical complications (I² ranging from 94% to 97%) warrants careful interpretation. This variability likely stems from differences in patient selection criteria, surgical complexity, institutional protocols for prophylaxis and monitoring, and varying definitions of complications across studies. The wide CIs for these outcomes suggest that larger, more homogeneous studies are needed to characterize these risks definitively.

Our meta-analysis, supported by the existing literature, highlights several key strategies to optimize outcomes in individuals with RA undergoing lumbar spine surgery. The first priority lies in thorough preoperative preparation. Adequate control of RA activity and associated comorbid conditions such as obesity, chronic obstructive pulmonary disease, and diabetes may lessen the likelihood of perioperative complications. Collaboration with rheumatology specialists is particularly important when considering adjustments to glucocorticoids or biologic DMARDs, balancing the potential for disease flare against heightened susceptibility to surgical site infection [28].

Attention to surgical detail also plays a major role. Gentle handling of tissues and avoiding unnecessarily prolonged operative time can be beneficial, given the propensity of RA patients to experience delayed wound healing. Extended postoperative antibiotic coverage remains a topic of discussion but may offer value in carefully selected individuals at elevated infectious risk. Notably, extended antimicrobial prophylaxis for up to seven days following prosthetic joint procedures has been associated with a reduction in periprosthetic joint infections among high-risk groups, including those living with RA, suggesting a potential parallel strategy for spine surgery [29].

The present study has certain limitations. All included studies were retrospective in design, introducing potential for selection bias and confounding. Second, substantial variability between studies evaluating reoperation and mortality outcomes limits the certainty of these pooled estimates. Even so, the overall trend of the effect measures indicates a potential association between RA and the primary endpoint. Third, none of the included investigations stratified outcomes by RA disease activity, leaving an important clinical question unanswered. Comparative analyses of patients with well-controlled vs. active RA could provide valuable insight into how inflammatory burden influences postoperative results. Finally, we were not able to perform subgroup analysis or meta-regression to identify factors associated with complications in RA subjects. Future studies are required to understand how different factors can affect the outcomes in RA subjects.

Future research should focus on prospective cohort studies with detailed characterization of RA disease activity, medication use, and bone quality at the time of surgery. Investigation of specific risk factors within the RA population that predict adverse outcomes would facilitate individualized risk stratification.

## Conclusions

This systematic review and meta-analysis demonstrates that RA significantly increases the risk of reoperation and surgical site infection following lumbar spine surgery, with RRs of 1.34 and 1.45, respectively. While mortality and major medical complications showed no statistically significant differences, the consistent elevation in surgical complications across diverse populations highlights the need for heightened perioperative vigilance in this vulnerable patient group. These findings emphasize the importance of comprehensive preoperative optimization, multidisciplinary collaboration with rheumatology specialists, and implementation of targeted infection prevention protocols. Surgeons should incorporate these risks into shared decision-making and preoperative counseling. Future prospective studies examining the impact of disease activity, specific immunosuppressive agents, and bone quality on surgical outcomes are essential to refine risk stratification and develop evidence-based perioperative management strategies for RA patients undergoing lumbar spine surgery.
